# Inflammatory Dendritic Cells, Regulated by IL-4 Receptor Alpha Signaling, Control Replication, and Dissemination of *Leishmania major* in Mice

**DOI:** 10.3389/fcimb.2019.00479

**Published:** 2020-01-24

**Authors:** Ramona Hurdayal, Natalie Eva Nieuwenhuizen, Rethabile Khutlang, Frank Brombacher

**Affiliations:** ^1^Department of Molecular and Cell Biology, University of Cape Town, Cape Town, South Africa; ^2^International Centre for Genetic Engineering and Biotechnology, Cape Town Component, Cape Town, South Africa; ^3^Division of Immunology, Department of Pathology, Faculty of Health Sciences, South African Medical Research Council on Immunology of Infectious Diseases, Institute of Infectious Diseases and Molecular Medicine, University of Cape Town, Cape Town, South Africa; ^4^Faculty of Health Sciences, Wellcome Centre for Infectious Diseases Research in Africa, Institute of Infectious Diseases and Molecular Medicine, University of Cape Town, Cape Town, South Africa; ^5^Department of Immunology, Max Planck Institute for Infection Biology, Berlin, Germany; ^6^Identity Authentication Research Group, Defence and Security, Council for Scientific and Industrial Research, Pretoria, South Africa

**Keywords:** *Leishmania major*, IL-4Rα, IL-4, dendritic cell, mice

## Abstract

Leishmaniasis is a vector-borne disease caused by *Leishmania* parasites. Macrophages are considered the primary parasite host cell, but dendritic cells (DCs) play a critical role in initiating adaptive immunity and controlling *Leishmania* infection. Accordingly, our previous study in CD11c^cre^IL-4Rα^−/lox^ mice, which have impaired IL-4 receptor alpha (IL-4Rα) expression on CD11c^+^ cells including DCs, confirmed a protective role for IL-4/IL-13-responsive DCs in replication and dissemination of parasites during cutaneous leishmaniasis. However, it was unclear which DC subset/s was executing this function. To investigate this, we infected CD11c^cre^IL-4Rα^−/lox^ and control mice with *L. major* GFP^+^ parasites and identified subsets of infected DCs by flow cytometry. Three days after infection, CD11b^+^ DCs and CD103^+^ DCs were the main infected DC subsets in the footpad and draining lymph node, respectively and by 4 weeks post-infection, Ly6C^+^ and Ly6C^−^ CD11b^+^ DCs were the main infected DC populations in both the lymph nodes and footpads. Interestingly, Ly6C^+^CD11b^+^ inflammatory monocyte-derived DCs but not Ly6C^−^CD11b^+^ DCs hosted parasites in the spleen. Importantly, intracellular parasitism was significantly higher in IL-4Rα-deficient DCs. In terms of DC effector function, we found no change in the expression of pattern-recognition receptors (TLR4 and TLR9) nor in expression of the co-stimulatory marker, CD80, but MHCII expression was lower in CD11c^cre^IL-4Rα^−/lox^ mice at later time-points compared to the controls. Interestingly, in CD11c^cre^IL-4Rα^−/lox^ mice, which have reduced Th1 responses, CD11b^+^ DCs had impaired iNOS production, suggesting that DC IL-4Rα expression and NO production is important for controlling parasite numbers and preventing dissemination. Expression of the alternative activation marker arginase was unchanged in CD11b^+^ DCs in CD11^cre^IL-4Rα^−/lox^ mice compared to littermate controls, but RELM-α was upregulated, suggesting IL-4Rα-independent alternative activation. In summary, *L. major* parasites may use Ly6C^+^CD11b^+^ inflammatory DCs derived from monocytes recruited to infection as “Trojan horses” to migrate to secondary lymphoid organs and peripheral sites, and DC IL-4Rα expression is important for controlling infection.

## Introduction

Leishmaniasis is a vector-borne parasitic infection caused by *Leishmania* species, obligate intracellular protozoans that are transmitted by the bite of infected female Phlebotominae sandflies. There are over 20 *Leishmania* species, and over 90 sandfly species known to transmit the parasites (Burza et al., 2018; WHO, 2019). According to the World Health Organization (WHO), ~700,000-1 million new cases and 26,000-65,000 deaths occur annually (WHO, 2019). Cutaneous leishmaniasis is the most common form of the disease, causing disfiguring, often ulcerative skin lesions. Mucocutaneous leishmaniasis leads to destruction of the mucous membranes of the nose, mouth, and throat, while visceral leishmaniasis involves dissemination of the parasites to organs, such as the spleen, liver, and bone-marrow, and is usually fatal if left untreated (Burza et al., 2018). While vector control remains an important component in controlling disease transmission, other efforts have focused on the design of novel drugs or vaccines against *Leishmania* species (Handman, 2001).

*Leishmania* parasites have two morphological stages: a flagellated promastigote form that is found in the salivary glands of the insect vector and a non-motile amastigote form that is found intracellularly in the vertebrate host (Gutiérrez-Kobeh et al., 2018). Experimental infections in mouse models have shown that promastigotes infect macrophages and neutrophils that are present at the site of inoculation (Sunderkotter et al., 1993; Laskay et al., 2003; Hurdayal et al., 2013; Gutiérrez-Kobeh et al., 2018). The primary host cell for *Leishmania* species is considered the macrophage, wherein the parasites differentiate into amastigotes and divide within parasitophorous vacuoles (Lievin-Le Moal and Loiseau, 2016). The release of new amastigotes causes the infection to spread. Parasite killing is dependent on IFN-gamma (IFN-γ)-mediated classical activation of macrophages to induce killing effector molecules, such as nitric oxide (NO) (Liew et al., 1990; Stenger et al., 1994; Diefenbach et al., 1999; Holscher et al., 2006). Immunity to leishmaniasis therefore depends on the production of IL-12, which drives T helper 1 (Th1) responses and the production of IFN-γ. However, infection with *Leishmania* downregulates the capacity of macrophages to produce IL-12 (Belkaid et al., 1998). Dendritic cells (DCs), on the other hand, produce IL-12 upon taking up amastigote parasites (Woelbing et al., 2006). At the same time, they mature, upregulate MHCII and co-stimulatory molecules, and travel to the lymph nodes (LN), where they prime naïve T cells to differentiate into Th1 cells, producing IFN-γ. DCs therefore play a critical role in initiating adaptive immunity and controlling *Leishmania* infection.

Interestingly, optimal induction of Th1 responses by DCs requires the Th2 cytokine IL-4, which paradoxically promotes IL-12 production by dendritic cells via inhibition of IL-10 (Biedermann et al., 2001; Lutz et al., 2002; Yao et al., 2005; Hurdayal et al., 2013). In a previous study, we found that CD11c^cre^IL-4Rα^−/lox^ mice, in which DCs lack IL-4Rα and are thus impaired in IL-4/IL-13 signaling, were hypersusceptible to cutaneous *L. major* infection in comparison to littermate control mice (Hurdayal et al., 2013). This mouse strain showed increased footpad swelling and necrosis, increased Th2 responses as well as substantially increased parasite burdens in LN, spleens and peripheral organs, such as the liver and even the brain. Importantly, we also found that DCs themselves harbored parasites, and that iNOS production was impaired in IL-4Rα deficient CD11c^hi^MHCII^hi^ DCs. The observation of infected DCs at peripheral sites suggested that DCs may play a role in disseminating *L. major*, and their effector responses could be important in controlling disease.

However, dendritic cells are recognized as a complex array of heterogeneous cell populations, and classified into different subsets by their surface markers, effector functions and ontogeny (Steinman and Inaba, 1999; Scott and Hunter, 2002; Zhou and Wu, 2017; Gutiérrez-Kobeh et al., 2018). Thus, we aimed to determine which subset of DCs is responsible for hosting and disseminating *L. major* parasites. We found that CD11b^+^Ly6C^+^ inflammatory DCs were most highly infected DC subset in CD11c^cre^IL-4Rα^−/lox^ mice, and that these DCs had impaired iNOS production in the absence of IL-4Rα signaling. This suggests that *L. major* parasites use inflammatory DCs as a “Trojan horse” to migrate to secondary lymphoid organs and peripheral sites, and that IL-4Rα signaling contributes to parasite control.

## Materials and Methods

### Generation and Genotyping of CD11c^cre^IL-4Rα^-/lox^ BALB/c Mice

Generation, characterization and genotyping of CD11c^cre^IL-4Rα^−/lox^ mice was performed as previously described (Hurdayal et al., 2013). Briefly, *Cd11c*^cre^ mice were inter-crossed with IL-4Rα^lox/lox^ BALB/c mice and homozygous IL-4Rα^−/−^ BALB/c mice (Mohrs et al., 1999) to generate hemizygous CD11c^cre^IL-4Rα^−/lox^ mice, backcrossed to a BALB/c background for nine generations to generate CD11c^cre^IL-4Rα^−/lox^ BALB/c mice. Hemizygous littermates (IL-4Rα^−/lox^) expressing functional IL-4Rα were used as wildtype controls in all experiments. All mice were housed in specific pathogen-free barrier conditions in individually ventilated cages at the University of Cape Town biosafety level 2 animal facility. Experimental mice were age and sex matched and used between 8 and 12 weeks of age.

### Ethics Statement

This study was performed in strict accordance with the recommendations of the South African national guidelines and University of Cape Town of practice for laboratory animal procedures. All mouse experiments were performed according to protocols approved by the Animal Research Ethics Committee of the Health Sciences Faculty, University of Cape Town (Permit Numbers: 009/042; 015/034). All efforts were made to minimize suffering of the animals.

### *Leishmania major* Infection

Green-fluorescent protein (GFP)-labeled *L. major* IL81 (MHOM/IL/81/FEBNI) (Gonzalez-Leal et al., 2014) strains were maintained by continuous passage in BALB/c mice and prepared for infection as described previously (Hurdayal et al., 2013). Anesthetized mice were inoculated subcutaneously with 2 × 10^6^ stationary phase promastigotes into the left hind footpad in a volume of 50 μl of sterile PBS. Swelling of infected footpads and weights of infected animals was monitored weekly using a Mitutoyo micrometer caliper (Brütsch, Zürich, Switzerland).

### Isolation of Footpad, Lymph Node, and Spleen Cells

Single lymph node cell suspensions were prepared by pressing the draining popliteal lymph nodes through 40 μM cell-strainers. Single cell suspensions of spleen cells were isolated by pressing spleens through 70 μM cell-strainers followed by red blood cell lysis. To isolate a single cell population from the infected footpad, footpads were treated with DMEM medium supplemented with Collagenase IV (Sigma-Aldrich; 1 mg/ml) and DNase I (Sigma-Aldrich; 1 mg/ml) at 37°C for 60 min to digest muscle and collagen. Following incubation, single cell footpad suspensions were isolated by straining through 40 μM cell-strainers. All cell suspensions of lymph node, spleen and footpad were resuspended in complete DMEM (Gibco) supplemented with 10% FCS (Gibco) and penicillin and streptomycin (100 U/ml and 100 μg/ml, Gibco) and enumerated using trypan blue exclusion (Hurdayal et al., 2013).

### Flow Cytometry

Antibodies against the following extracellular markers were used for flow cytometry: CD11c, CD11b, MHCII, F4/80, CD3, CD4, CD19, CD103, and Ly6C (all BD Bioscience, Erembodegem, Belgium). Footpad, pLN, and spleen cells (1 × 10^6^) were stained with antibody cocktails in PBS containing 1% BSA/1% rat serum for 20 min at 4°C followed by fixing in 2% [w/v] PFA. For intracellular cytokine staining of dendritic cells, popliteal lymph node cells from *L. major* infected mice were seeded at 2 × 10^6^ cells/well, surface-stained, fixed and permeabilized, and stained intracellularly for iNOS using rabbit anti-mouse iNOS (Abcam) with goat anti-rabbit PE (Abcam) and goat anti-mouse arginase (Santa Cruz Biotechnology) with donkey anti-goat PE (Abcam). Staining specificity was verified by isotype-matched antibody controls and compensation performed with BD compensation beads. Acquisition was performed using BD LSRFortessa (BD Biosciences), and data were analyzed using FlowJo software (Treestar, Ashland, OR, USA).

### *Ex vivo* Restimulation of Footpad and Lymph Node Cells

Footpad and lymph node cells, resuspended in complete DMEM (Gibco) supplemented with 10% FCS (Gibco) and penicillin and streptomycin (100 U/ml and 100 μg/ml, Gibco) were cultured at 1 × 10^6^ cells in 48-well plates together with 50 μg/ml soluble *Leishmania* antigen (SLA). Cells were incubated at 37°C in a humidified atmosphere containing 5% CO_2_. Supernatants were collected after 72 h and stored at −80°C for cytokine analysis.

### Enzyme-Linked Immunosorbent Assay (ELISA)

Cytokines (IL-4, IFN-γ, and TNF-α) in supernatants from restimulated footpad or lymph node cells were measured by indirect sandwich ELISA as previously described (Mohrs et al., 1999; Hurdayal et al., 2013).

### Confocal Microscopy

OCT-embedded lymph node and spleen tissue from mice infected with GFP-*L. major* IL81 parasites for 4 weeks was cut into 10 μm cryosections. Following acetone fixation, dendritic cells were stained using biotinylated anti-CD11c mAb (BD Biosciences) and visualized by staining with a streptavidin-Cy3 conjugate (Sigma). Nuclei were stained with Hoechst. Coverslips were then mounted on sections using Mowiol®4-88 mounting medium (Calbiochem) with anti-fade (Sigma). Images were acquired by Ziess LSM 510 confocal microscope and images quantified using a MatLab (MathWorks, Natick, Massachusetts) script developed for automated counting. A total of 16 fields were captured for each condition. At week 4 after GFP-*L. major* IL81 infection, lymph node B cells (CD19^+^CD3^−^CD11c^−^) were isolated by cell sorting on a FACS Vantage cell sorter. Sorted cells were viewed live, directly in suspension in chamber slides for the presence of GFP^+^ parasites by LSM 510 confocal microscopy.

### Statistics

Data is given as mean ± SEM. Statistical analysis was performed using the unpaired Student's *t*-test or 1-way Anova with Bonferroni's post-test, defining differences to IL-4Rα^−/lox^ mice as significant (^*^p ≤ 0.05; ^**^p ≤ 0.01; ^***^p ≤ 0.001) (Prism software; https://www.graphpad.com/scientific-software/prism/).

## Results

### Ly6C^+^CD11b^+^ DCs Are the Predominant Infected DC Subset Associated With Dissemination in *L. major* Infected Mice

**Figure 1:**
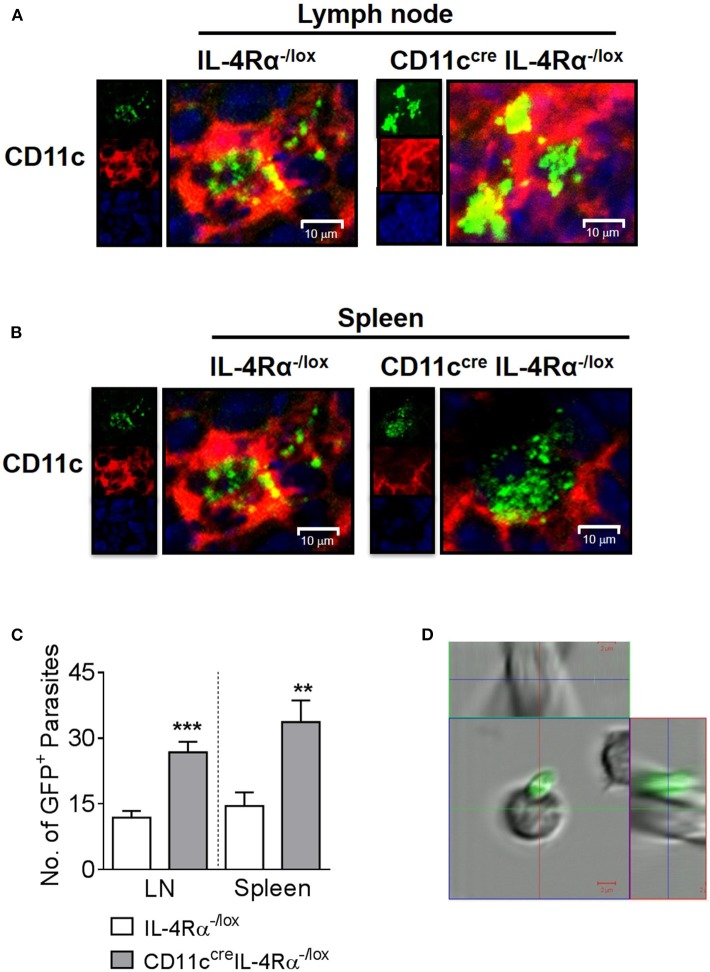
Intracellular nature of GFP^+^ *L. major* parasites in dendritic cells of lymph nodes and spleen. Mice were infected subcutaneously with 2 × 10^5^ stationary phase GFP-expressing *L. major* IL81 promastigotes into the hind footpad. After 4 weeks of infection, frozen sections of lymph node and spleen were stained with a mAb against CD11c^+^ dendritic cells. Representative micrographs of lymph node **(A)** and spleen cryosections **(B)** showing intracellular localization of GFP^+^ *L. major*-infected (green) CD11c^+^ dendritic cells (red) from CD11c^cre^IL-4Rα^−/lox^ and littermate mice (original magnification ×400). Insets show individual channels. The actual number of GFP^+^ amastigote parasites in dendritic cells from CD11c^cre^IL-4Rα^−/lox^ and littermate control mice were quantified using MatLab software in 16 fields of multiple lymph node and spleen sections **(C)** from individual mice by confocal microscopy. At the same time-point, lymph node B cells (CD19^+^CD3^−^CD11c^−^) were isolated on a FACS Vantage cell sorter after staining with specific mAbs **(D)**. Live sorted cells were viewed directly in suspension in chamber slides for the location of GFP^+^ parasites by LSM 510 confocal microscopy. Data is expressed as mean ± SEM. Statistical analysis was performed defining differences to IL-4Rα^−/lox^ mice (**p ≤ 0.01; ***p ≤ 0.001) as significant.

Previously, we found increased numbers of DCs containing GFP-expressing *L. major* parasites in CD11c^cre^IL-4Rα^−/lox^ mice compared to littermate controls using flow cytometry (Hurdayal et al., 2013). To confirm the presence of intracellular parasites in DC subsets by fluorescent imaging, CD11c^cre^IL-4Rα^−/lox^ and control mice were infected with GFP-expressing *L. major* in the hind footpad. As found previously (Hurdayal et al., 2013), by week 4 post-infection, CD11c^cre^IL-4Rα^−/lox^ mice presented with fulminant cutaneous leishmaniasis, and were hypersusceptible compared to littermate control mice (Supplementary Figure S1). Representative confocal images of the pLN (Figure 1A) and spleen (Figure 1B) demonstrate the intracellular amastigote nature of most of the GFP^+^ parasites in CD11c^+^ cells, indicating that these cell populations were harboring whole parasites and not fragments of GFP. Automated quantification of intracellular GFP^+^ *L. major* amastigote parasites in the cryo-fixed tissue sections using MatLab software revealed significantly increased numbers of parasites per cell in CD11c^+^ cells of CD11c^cre^IL-4Rα^−/lox^ mice compared to littermate control mice in both the lymph node and spleen (Figure 1C) in accordance with previous flow cytometry data (Hurdayal et al., 2013). In contrast, confocal imaging of FACS-sorted B cells demonstrated GFP^+^ parasites adhering to the outside of the cells and not intracellularly (Figure 1D) confirming that lymphocyte populations do not support replication of intracellular *Leishmania*. This illustrates the importance of confirming that GFP^+^ cells actually harbor intracellular pathogens. Altogether, this strengthens and confirms previous data, in which CD11c-expressing cells served as a reservoir for pathogen replication (Heyde et al., 2018), and showed increased infection in the absence of IL-4-signaling via the IL-4Rα chain (Hurdayal et al., 2013).

**Figure 2:**
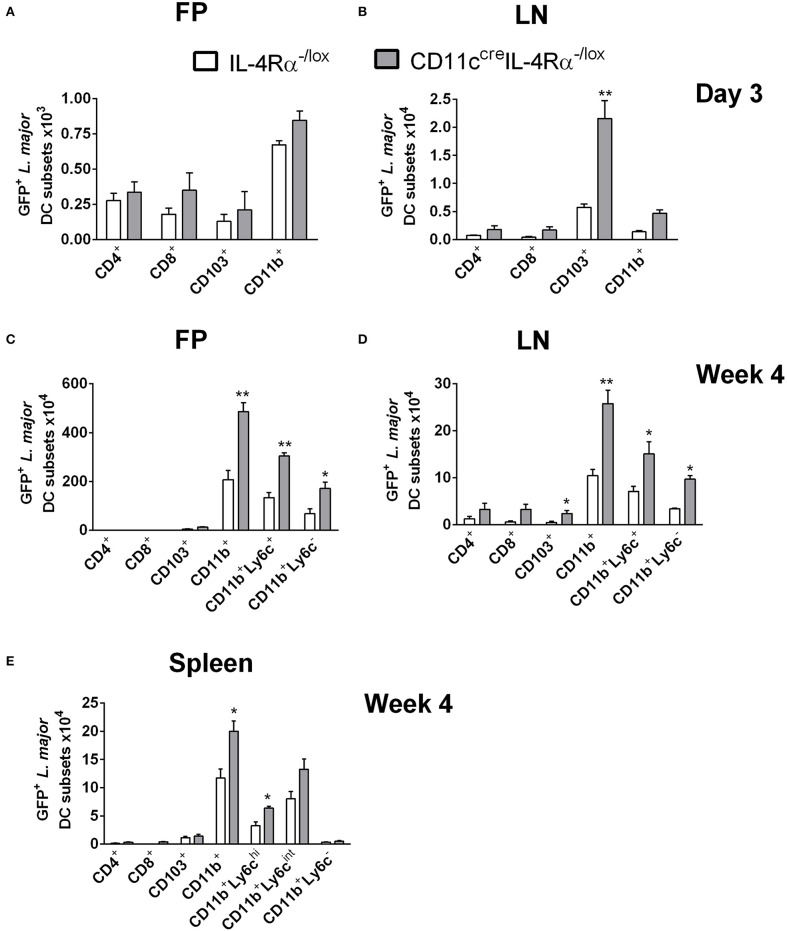
CD11b^+^ inflammatory dendritic cells in CD11c^cre^IL-4Rα^−/lox^ mice preferentially harbor *L. major* parasites during infection. CD11c^cre^IL-4Rα^−/lox^ and littermate mice were infected subcutaneously with GFP-labeled *L. major* IL81 promastigotes into the hind footpad. Number of GFP^+^ *L. major* parasites was identified within dendritic cell subsets derived from footpad **(A,C)** and lymph node **(B,D)** at day 3 and week 4 after infection, respectively. Spleens were harvested to analyse GFP^+^ *L. major* parasites in the indicated DC subsets **(E)** at week 4 after infection. Dendritic cell populations were gated as shown in Supplementary Figure S1 and differentiated based on the following markers; conventional lymphoid-derived dendritic cells; CD4^+^ DCs (CD11c^+^CD4^+^CD3^−^), CD8^+^ DCs (CD11c^+^CD8^+^CD3^−^), and myeloid-derived dendritic cells; CD103^+^ DCs (CD11c^+^MHCII^+^CD103^+^), CD11b^+^ DCs (CD11c^+^CD11b^+^MHCII^+^), monocyte-derived DCs (CD11c^+^CD11b^+^MHCII^+^Ly6C^+^), and non-monocyte derived DCs (CD11c^+^CD11b^+^MHCII^+^Ly6C^−^). Statistical analysis was performed defining differences to IL-4Rα^−/lox^ mice (*, *p* ≤ 0.05; **, *p* ≤ 0.01) as significant.

Since DCs represent a highly heterogeneous cell population comprising various subsets defined by their effector functions and surface markers (Steinman and Inaba, 1999; Scott and Hunter, 2002; Zhou and Wu, 2017), we sought to investigate if any particular DC subset was important in harboring and disseminating *L. major* in infected mice. To determine which subsets harbor *L. major* after infection, CD11c^cre^IL-4Rα^−/lox^ and littermate control mice were infected in the footpads as above and infected cell populations were tracked by flow cytometry at early (day 3) and late (week 4) stages of infection. DC subsets were identified by their expression of particular cell-surface markers, focusing on the lymphoid and myeloid DC subsets. In line with their limited phagocytic ability (Pulendran et al., 1997), lymphoid-resident DCs (CD4^+^CD11c^hi^CD3^−^ and CD8^+^CD11c^hi^CD3^−^) DCs were the least infected DC population, both at day 3 and at week 4 post-*L. major* infection in both CD11c^cre^IL-4Rα^−/lox^ and littermate control mice. Myeloid-derived DCs are a migratory subset of conventional DCs, known to take up antigen at the infection site and migrate to the draining LN for presentation to antigen-specific T cells, with two major subsets being CD103^+^ migratory tissue DCs and CD11b^+^ DCs (Pulendran et al., 1997; Steinman and Inaba, 1999; Martinez-Lopez et al., 2015; Mayer et al., 2017). At day 3 post-*L. major* infection, CD11b^+^ DCs (Supplementary Figure S2) were the main subset infected with *L. major* in the footpads of both CD11c^cre^IL-4Rα^−/lox^ mice and littermate controls (Figure 2A). However, in the LN of CD11c^cre^IL-4Rα^−/lox^ mice there was an increased number of GFP^+^ CD103^+^ DCs that had most likely carried *L. major* parasites from the tissue to the LNs (Supplementary Figure S2 and Figure 2B). CD11b^+^ DCs can be further divided into monocyte-derived/inflammatory DCs or non-monocyte derived, with monocyte-derived DCs (mo-DCs) typically expressing Ly6C and induced as part of the inflammatory response as infection progresses (Leon et al., 2007; Plantinga et al., 2013). Thus, at the later stage of infection (week 4), we included Ly6c as a marker to differentiate monocyte-derived DCs and non-monocyte derived DCs (Supplementary Figure S2). CD11b^+^ DCs (both Ly6C^+^ and Ly6C^−^) were the main infected DC populations in both the footpads and lymph nodes at week 4 post-infection, and numbers of infected CD11b^+^ DCs were greater in CD11c^cre^IL-4Rα^−/lox^ mice compared to littermate controls (Figures 2C,D). This indicates that monocyte-derived DCs that were recruited to the footpad also became infected with *L. major* parasites.

While it is known that parasites migrate from the site of infection to the lesion-draining lymph nodes, we were interested to determine if *L. major* parasites were using CD11b^+^ Mo-DCs or CD11b^+^ cDCs to disseminate to peripheral organs, and if IL-4/IL-13 signaling influenced this process. Therefore, we analyzed infected DC subsets in the spleens of CD11c^cre^IL-4Rα^−/lox^ and littermate control mice at 4 weeks post-*L. major* infection. The results showed that by week 4 post-infection, CD11b^+^ DCs were the main subset containing GFP^+^ *L. major* in the spleen (Figure 2E). Ly6C^+^ CD11b^+^ DCs consisted of two populations, Ly6C high (Ly6C^hi^) and Ly6C intermediate (Ly6C^int^). Both Ly6C^hi^ and Ly6C^int^ populations harbored intracellular *Leishmania*. In contrast, Ly6C^−^ CD11b^+^ DCs did not appear to be host cells for intracellular parasites. Importantly, the numbers of infected CD11b^+^ DCs in FPs, LNs and spleens were higher in CD11c^cre^IL-4Rα^−/lox^ mice, suggesting a requirement for IL-4/IL-13 signaling in controlling dissemination of intracellular *Leishmania*. Taken together, these data suggest that *L. major* parasites utilize inflammatory CD11b^+^Ly6C^+^ DCs as “Trojan horses” to migrate to secondary lymphoid organs and that IL-4Rα-signaling contributes to parasite control in these cells.

### Phenotype of CD11b^+^ Dendritic Cells in CD11c^cre^IL-4Rα^-/lox^ Mice During *L. major* Infection

**Figure 3:**
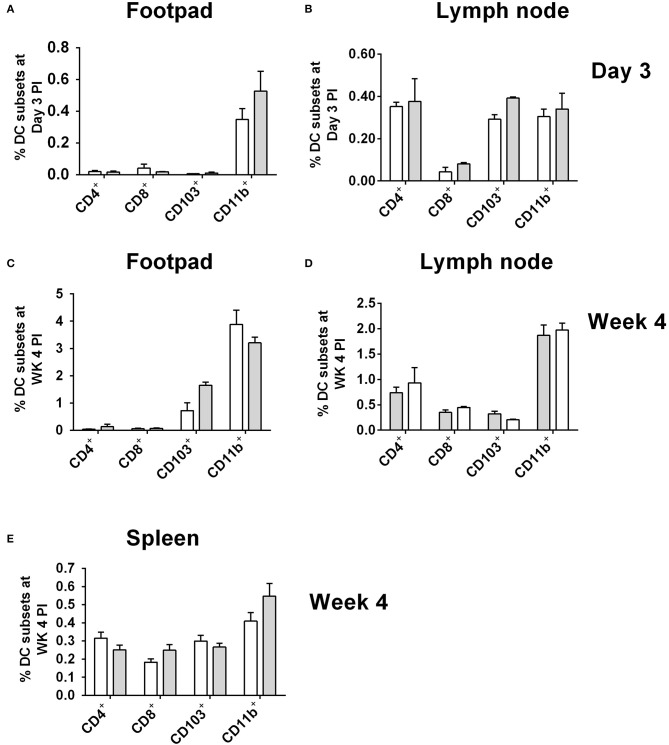
Frequency of dendritic cells in during *L. major* IL81 infection in CD11c^cre^IL-4Rα^−/lox^ mice. CD11c^cre^IL-4Rα^−/lox^ and littermate mice were infected subcutaneously with GFP-labeled *L. major* IL81 promastigotes into the hind footpad. At day 3 and week 4 after infection, total footpad **(A,C)**, lymph node **(B,D)**, and spleen **(E)** cells were surface stained for total frequency of dendritic cells subsets. Cell populations were differentiated based on the following markers; conventional lymphoid-derived dendritic cells; CD4^+^ DCs (CD11c^+^CD4^+^CD3^−^), CD8^+^ DCs (CD11c^+^CD8^+^CD3^−^), and myeloid-derived dendritic cells; CD103^+^ DCs (CD11c^+^MHCII^+^CD103^+^), CD11b^+^ DCs (CD11c^+^CD11b^+^MHCII^+^), monocyte-derived DCs (CD11c^+^CD11b^+^MHCII^+^Ly6C^+^), and non-monocyte derived DCs (CD11c^+^CD11b^+^MHCII^+^Ly6C^−^).

As CD11b^+^ DCs were the most highly infected DC subset in CD11c^cre^IL-4Rα^−/lox^ mice at day 3 (footpad) and week 4 post-*L. major* infection, we checked whether the higher numbers of infected cells could be due to increased numbers of these cells overall, as a consequence of IL-4Rα-deficiency. At day 3 post-infection with GFP-expressing *L. major* IL81 parasites, similar levels of all DC subsets, including CD11b^+^ DCs, was found in infected footpad (Figure 3A) and lymph node (Figure 3B). As there was no dissemination to the spleen at Day 3 post-infection (Hurdayal et al., 2013), analysis of DC subsets in the spleen was not performed at this time-point. The same trend in DC numbers was seen at week 4 post-infection, with similar numbers of all DC subsets, including CD11b^+^ DCs, infiltrating the FP (Figure 3C), LN (Figure 3D), and spleen (Figure 3E) in CD11c^cre^IL-4Rα^−/lox^ mice and littermate controls. Moreover, analysis of CD11b^+^ DCs in the spleen of naïve animals and at Day 1 and 3 found no difference in infiltration of CD11b^+^ DCs between CD11c^cre^IL-4Rα^−/lox^ mice and littermate controls (Supplementary Figure S3). This suggests that altered effector functions of CD11b^+^ DCs, and not increased numbers, contributed to increased parasites loads and dissemination in the absence of IL-4Rα signaling.

**Figure 4:**
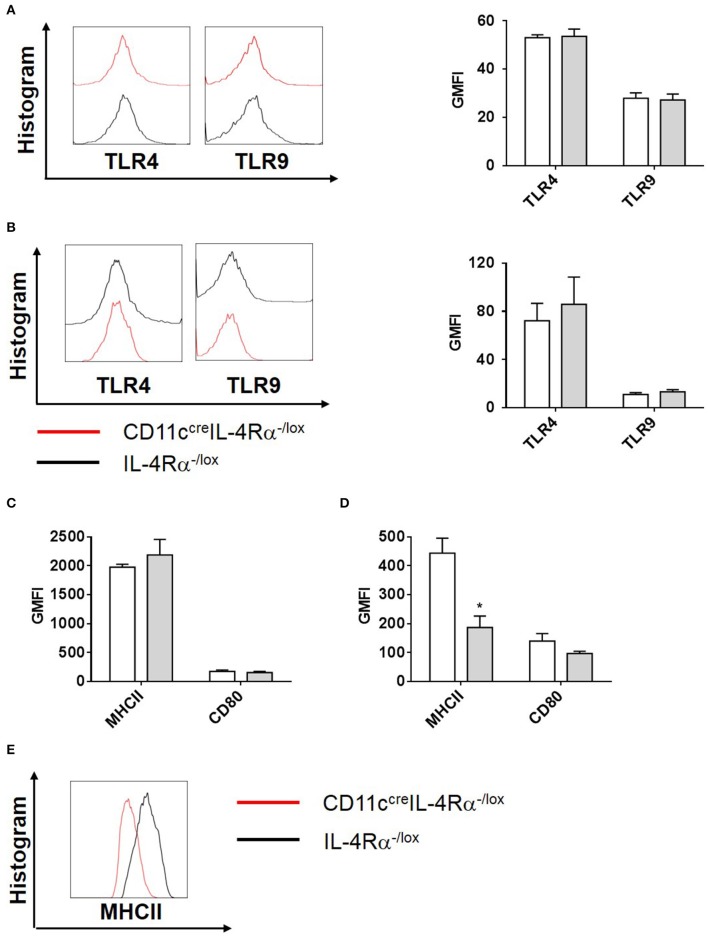
Phenotype of CD11b^+^ DCs during *L. major* infection in CD11c^cre^IL-4Rα^−/lox^ mice and controls. CD11c^cre^IL-4Rα^−/lox^ and littermate mice were infected subcutaneously with GFP-labeled *L. major* IL81 promastigotes into the hind footpad. At week 4 after infection, total footpad cells were stained for levels of pattern-recognition receptors, TLR4 and TLR9 on CD11b^+^ DCs at day 3 **(A)** and week 4 **(B)** after infection. Similarly, activation markers (MHCII and CD80) on CD11c^+^MHCII^+^CD11b^+^ DCs in the footpad were analyzed at day 3 **(C)** and week 4 **(D)** after infection. Histogram plots of MHCII expression at week 4 after infection **(E)**. Statistical analysis was performed defining differences to IL-4Rα^−/lox^ mice (*, *p* ≤ 0.05) as significant.

**Figure 5:**
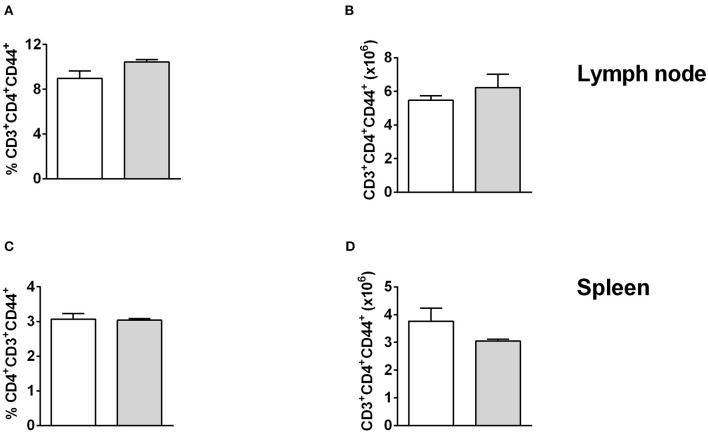
T cell activation is unchanged in lymph node and spleen of CD11c^cre^IL-4Rα^−/lox^ mice and controls. CD11c^cre^IL-4Rα^−/lox^ and littermate mice were infected subcutaneously with GFP-labeled *L. major* IL81 promastigotes into the hind footpad. At week 4 after infection, total cells were stained for the frequency **(A,C)** and absolute number **(B**, **D)** of effector T helper cells (CD3^+^CD4^+^CD44^+^) in the lesion-draining popliteal lymph node **(A,B)** and spleen **(C,D)**.

To investigate this further, we analyzed the effector functions of CD11b^+^ DCs at early and late stages after *L. major* infection in CD11c^cre^IL-4Rα^−/lox^ and control mice. DC activation, differentiation into subsets and subsequent parasite control has been linked to antigen recognition via pattern-recognition receptors (PRRs), specifically the Toll-like receptors (TLRs) (Faria et al., 2012). Since TLR4 and TLR9 are reportedly required for mounting an effective Th1 response (Faria et al., 2012) and hypersusceptible CD11c^cre^IL-4Rα^−/lox^ mice showed a shift toward Th2 responses in *L. major* infection (Hurdayal et al., 2013), we evaluated expression of these receptors on CD11b^+^ DCs by gating CD11c^+^CD11b^+^MHCII^+^ populations and analyzing mean fluorescence intensity of histograms of expression levels (Supplementary Figure S2 and Figure 4). At Day 3 post-infection in the footpad, CD11b^+^ DCs from CD11c^cre^IL-4Rα^−/lox^ and littermate control mice expressed equivalent levels of TLR4 and TLR9 (Figure 4A). A similar trend was observed at week 4 post-infection in the footpad (Figure 4B). Once DCs are activated by pathogen products, they mature to express higher levels of MHCII and costimulatory molecules, such as CD80 and CD86, and can present antigen to prime naïve T cells (Pulendran et al., 1997), therefore we measured MHCII and CD80 expression on CD11b^+^ DCs in the lesion-draining lymph node. We and others have reported previously that lack of IL-4Rα signaling on DCs does not intrinsically alter expression of MHCII or co-stimulatory molecules, such as CD80, CD86, and CD40 *in vivo* (Cook et al., 2012; Hurdayal et al., 2013). At day 3 post-infection, CD11b^+^ DCs from CD11c^cre^IL-4Rα^−/lox^ and littermate control mice showed equivalent expression of MHCII and CD80 (Figure 4C), demonstrating that early activation of DCs was unaltered in the absence of IL-4Rα expression. At later time-points (week 4 post-infection), MHCII expression was lower in the absence of IL-4Rα on DCs (Figures 4D,E) whilst CD80 expression was similar between CD11c^cre^IL-4Rα^−/lox^ and littermate control mice (Figure 4D). Nevertheless, lower levels of MHCII expression did not correspond to differences in ability of DCs to prime differentiation of naïve Th cells into effector/memory T cells, since similar percentages and numbers of CD4^+^ T cells (gated FSC^low^SSC^low^CD3^+^CD4^+^ as described in Hurdayal et al., 2017) were CD44^+^ in the LN of CD11c^cre^IL-4Rα^−/lox^ mice and littermate controls (Figures 5A,B). Similarly, we found similar levels of CD4^+^CD44^+^ T cells in the spleens of CD11c^cre^IL-4Rα^−/lox^ and littermate control mice (Figures 5C,D). Taken together, these data suggest that IL-4Rα might play a role in regulation of DC maturation at the later stages of infection, but is not required for T cell activation in the lesion-draining lymph node.

### IL-4Rα Regulates the Activation of CD11b^+^ DCs

**Figure 6:**
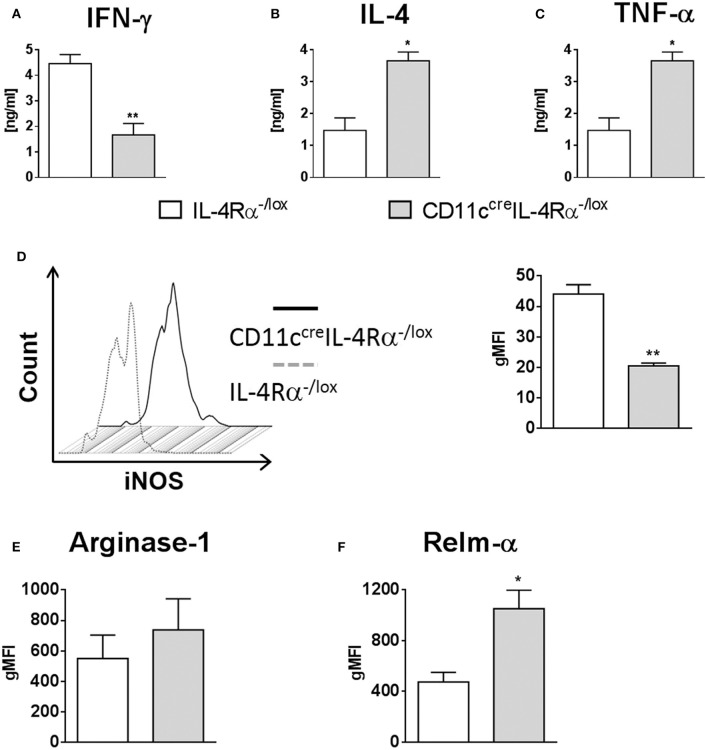
IL-4Rα regulates the activation of CD11b^+^ DCs during *L. major* infection in CD11c^cre^IL-4Rα^−/lox^ mice and controls. CD11c^cre^IL-4Rα^−/lox^ and littermate mice were infected subcutaneously with GFP-labeled *L. major* IL81 promastigotes into the hind footpad. At week 4 after infection, total footpad cells were re-stimulated with soluble *Leishmania* antigen (SLA) for 72 h and levels of IFN-γ **(A)** and IL-4 **(B)** was determined in cell supernatants by ELISA. TNF-α **(C)** was determined in draining lymph-node cells by ELISA following SLA stimulation. To analyse classical and alternate activation of DCs, CD11b^+^ DCs of total footpad cells were stained for intracellular levels of inducible nitric oxide synthase (iNOS) **(D)**, arginase **(E)**, and resistin-like molecule alpha-RELM-α **(F)** at week 4 after *L. major* infection. Statistical analysis was performed defining differences to IL-4Rα^−/lox^ mice (*, *p* ≤ 0.05; **, *p* ≤ 0.01) as significant.

Once phagocytosed, *Leishmania* parasites are eliminated by iNOS-induced nitric oxide production, which occurs predominantly in classically-activated macrophages in response to IFN-γ. In contrast, IL-4/IL-13 signaling via the IL-4Rα drives alternative activation of macrophages and arginase production, and this promotes parasite survival (Hurdayal and Brombacher, 2017). We measured cytokine production by cells from FPs of *L. major* infected mice restimulated with SLA, and found that IFN-γ production was significantly reduced in CD11c^cre^IL-4Rα^−/lox^ mice compared to littermate controls, whilst IL-4 production was dramatically increased, demonstrating a switch toward Th2-type responses at the site of infection (Figures 6A,B). Given the importance of inflammatory DCs in our model, we looked at production of the pro-inflammatory cytokine TNF-α by restimulated LN cells. Production of TNF-α was significantly higher in CD11c^cre^IL-4Rα^−/lox^ mice compared to those of littermate controls (Figure 6C). It has been shown that DCs may also be alternatively activated in schistosomiasis (Cook et al., 2012), and previously we found decreased iNOS in conventional DCs in spleens of *L. major* infected mice (Hurdayal et al., 2013). Therefore, we aimed to determine how IL-4Rα signaling affects the killing-effector phenotype of CD11b^+^ DCs in *L. major* infection. Intracellular staining of CD11b^+^ DCs in infected FPs demonstrated that intracellular iNOS expression was significantly lower in CD11b^+^ DCs of CD11c^cre^IL-4Rα^−/lox^ compared to those of littermate controls (Figure 6D). However, expression of the alternative activation marker arginase was unchanged in CD11b^+^ DCs in CD11c^cre^IL-4Rα^−/lox^ mice compared to littermate control mice (Figure 6E). Interestingly, RELMα, another marker associated with alternative activation of macrophages (Gordon, 2003) and DCs (Cook et al., 2012), was upregulated in CD11c^cre^IL-4Rα^−/lox^ mice (Figure 6F), suggesting IL-4/IL-4Rα-independent expression of RELMα on CD11b^+^ DCs.

## Discussion

Macrophages are considered the principal host cells of *Leishmania* parasites, and are key players in the parasite cycle of replication, release and entry into new host cells (Martinez-Lopez et al., 2018). However, dendritic cells can also be infected and appear to play an important role in disease progression (De Trez et al., 2009; Hurdayal et al., 2013). Dendritic cell-derived IL-12 is essential for the polarization of naïve T cells toward a Th1 subset and subsequent production of IFN-γ to control infection (Biedermann et al., 2001). In contrast, DC-derived IL-10 has been shown to dampen this process, leading to an environment that favors parasite replication and polarization of naïve T cells toward a Th2 subset (Yao et al., 2005). We previously demonstrated *in vivo* that IL-4 can instruct DCs to promote Th1 differentiation and effector iNOS production for killing of intracellular *L. major*, by using the cre/*loxP* recombination system to generate BALB/c mice deficient in the IL-4Rα gene under control of the *cd11c* locus (Hurdayal et al., 2013). In addition, we discovered that DCs function as important reservoirs for intracellular parasite replication and dissemination from the site of infection in the footpad.

DCs comprise a highly heterogeneous cell population consisting of various subsets with different characteristics (myeloid, lymphoid, migratory, plasmacytoid, and inflammatory) (Steinman and Inaba, 1999; Scott and Hunter, 2002; Zhou and Wu, 2017). They can be divided into three main groups: conventional DCs (cDCs), plasmacytoid DCs (pDCs), and monocyte-derived DCs (mo-DCs). DC-*Leishmania* interactions can vary depending on the different DC subsets involved, as they are equipped with different effector functions in terms of pathogen recognition, signal transduction and cytokine release. DCs are important for parasite uptake (via Toll-like receptors), processing, presentation, and subsequent activation of naïve T cells in adaptive immunity. They not only engulf apoptotic neutrophils harboring intracellular parasites but also engulf free extracellular promastigotes (Martinez-Lopez et al., 2018). It has also been shown that they can function as effector cells in killing of intracellular pathogens by expression of iNOS (De Trez et al., 2009; Hurdayal et al., 2013). Moreover, cytokine signaling has been shown to influence the effector function of DCs (Marovich et al., 2000; Girard-Madoux et al., 2015; Martinez-Lopez et al., 2015). After finding that mice with impaired IL-4Rα signaling on CD11c^+^ cells (CD11c^cre^IL-4Rα^−/lox^ mice) were susceptible to *L. major* and showed increased numbers of infected DCs (Hurdayal et al., 2013), we wanted to determine which DC subset, regulated by IL-4, might be important for intracellular multiplication and the spread of *L. major* parasites during infection, and which effector functions were involved in the phenotype. To address this, we used GFP-labeled parasites to track infected DC populations and flow cytometry to identify subsets of dendritic cells infiltrating the footpad, LN and spleen during the early (Day 3) and late stages (week 4) of *L. major* infection.

During early stages of infection in the skin, recruited monocytes are believed to differentiate into effector cells for uptake, processing and transport of antigen to the draining LN. It is therefore not surprising that migratory CD103^+^ DCs, which are derived from monocytes (Jakubzick et al., 2008; Del Rio et al., 2010), were the main source of infected cells in the draining popliteal LN at the early stage (day 3) of infection. CD103^+^ DCs are reported to be the main source of IL-12 upon infection with *L. major* parasites, important in inducing local Th1 immunity (Martinez-Lopez et al., 2015). Interestingly, the absence of IL-4/IL-13 signaling in these cells increased the percentage of cells that contained parasites, while the number of CD103^+^ DCs remained similar, suggesting that IL-4/IL-13 signaling to CD103^+^ migratory DCs could represent an early control mechanism for establishment of infection in the LN. A previous study also reported that DCs were the primary infected cell population in the draining LN of *L. major* infected mice, but did not examine DC subsets (Muraille et al., 2003). Our study expands upon these findings by demonstrating that CD103^+^ tissue DCs may be responsible for trafficking parasites to the draining LN at the onset of infection. This concept is supported by a study involving influenza virus infection in the lung, which demonstrated that CD103^+^ DCs were the cells that carried intact viral protein to the draining LNs, and that this occurred as early as 12 h after inoculation, peaking at 48 h after infection (Helft et al., 2012). Previously we showed that pDCs also harbor parasites at the site of infection early (day 3) after parasite inoculation (Hurdayal et al., 2013).

During the later stage of infection (week 4), we found that CD11b^+^ DCs, consisting of both monocyte-derived inflammatory DCs (Ly6C^+^) (Leon et al., 2007) and conventional DCs (Ly6C), were the main infected cells in both the FP and LN of BALB/c mice infected with *L. major*. Similar to what was seen with the CD103^+^ DCs, the frequency of parasite-infected CD11b^+^ DCs increased strikingly in the absence of IL-4-responsivness, suggesting that IL-4Rα-signaling on DCs contributes to intracellular parasitism of DCs themselves. In fact, all DCs subsets analyzed had increased parasite loads in the absence of IL-4Rα on DCs, while numbers of DCs infiltrating tissues were similar between CD11c^cre^IL-4Rα^−/lox^ mice and littermate controls. This suggests that differences in DC effector functions were responsible for the increased parasite replication and dissemination seen during infection. Our analysis demonstrated that hypersusceptibility, in the absence of IL-4-responsive DCs during *L. major* infection was not a consequence of altered expression of pattern recognition receptors (TLR4, TLR9) or expression of the co-stimulatory marker CD80. DC-derived MHCII levels were similar in CD11c^cre^IL-4Rα^−/lox^ mice and littermate controls at day 3, but at week 4 they were decreased in CD11c^cre^IL-4Rα^−/lox^ mice, although activation of Th cells as measured by CD44 expression was not affected. At week 4 in the spleen, *L. major* parasites were found primarily in Ly6C^+^CD11b^+^ DCs whereas Ly6C^−^CD11b^+^ DCs were minimally infected. This suggests that *L. major* uses Ly6C^+^ monocyte-derived inflammatory CD11b^+^ DCs to disseminate from primary LN organs during infection. This is in line with a previous study during *Mycobacterium tuberculosis* infection, in which activated inflammatory DCs (CD11c^+^CD11b^+^Ly6C^+^ DCs) showed a unique tendency to disseminate to systemic sites (Schreiber et al., 2011). In combination with the impaired ability to kill parasites because of reduced iNOS expression in these DCs, this would explain how CD11c^cre^IL-4Rα^−/lox^ mice were more susceptible to dissemination of *L. major*.

The term “classical activation” is characteristically employed for IFN-dependent activation of macrophages and secretion of nitric oxide to kill intracellular pathogens, such as *L. major* (Gordon, 2003; Gordon and Pluddemann, 2017). However, the combined evidence of several studies demonstrates that DCs can also express iNOS in order to control intracellular parasites (Serbina et al., 2003; De Trez et al., 2009; Hurdayal et al., 2013). We demonstrated previously that iNOS expression was decreased in CD11c^+^ DCs from CD11c^cre^IL-4Rα^−/lox^ mice compared to littermate controls, most likely as a consequence of decreased Th1 responses due to lack of IL-4 instruction of DCs (Biedermann et al., 2001; Hurdayal et al., 2013). Now we have expanded on this, showing that IL-4Rα-deficient CD11b^+^ DCs secrete decreased iNOS, which could play a significant role in parasite replication and indicates impaired classical activation. iNOS and arginase share the substrate L-arginine, and in macrophages, alternative activation of macrophages via IL-4Rα signaling leads to upregulation of arginase-1, which can promote parasite growth by arginase-dependent synthesis of polyamines (Kropf et al., 2005). Alternative activation of DCs by IL-4/IL-4Rα has been documented, in which DCs upregulate markers, such as RELM-α and Ym1/2 (Cook et al., 2012), However, DCs of CD11c^cre^IL-4Rα^−/lox^ mice have impaired responsiveness to IL-4/IL-13. Therefore, given the impaired classical activation, we examined markers of alternative activation to see whether they were affected by the loss of DC IL-4Rα signaling. Interestingly, we found that arginase-1 expression was unchanged in CD11c^cre^IL-4Rα^−/lox^ mice compared to littermate control mice, consistent with previous work showing that IL-4 does not modulate arginase-1 expression in murine DCs (Cook et al., 2012). Surprisingly however, we found that RELM-α, another marker of alternate activation (Cook et al., 2012), was upregulated in DCs of CD11c^cre^IL-4Rα^−/lox^ mice compared to littermate controls. This suggests that IL-4/IL-13-independent alternative activation of DCs can occur during *L. major* infection. Recent studies have demonstrated other mechanisms of alternative activation of macrophages. For example, interleukin-4-indiced gene 1 (IL-4I1) has been defined as a strong modulator of alternative macrophage activation (Yue et al., 2015), and is also expressed on DCs (Aubatin et al., 2018).

Suppression of monocyte function can also occur by the IFN-induced tryptophan-catabolizing enzyme indoleamine 2,3-dioxygenase (IDO) (Musso et al., 1994). Activated IDO depletes tryptophan levels and this depletion induces cell cycle arrest of T cells, ultimately increasing their apoptosis and causing immunosuppressive effects (Bilir and Sarisozen, 2017). It has been shown that IL-4 can inhibit IDO expression (Musso et al., 1994), so in the absence of responsiveness to IL-4, it is possible that DCs of CD11c^cre^IL-4R^α−/*lox*^ mice would have increased IDO expression, resulting in a more suppressive phenotype. Support for this hypothesis comes from the observation of increased TNF-α production by LN cells of CD11c^cre^IL-4Rα^−/lox^ mice compared to controls, as TNF-α has been shown to activate IDO secretion (Braun et al., 2005; Kim et al., 2015; Bilir and Sarisozen, 2017). However, the role of IDO remains to be investigated in hypersusceptible CD11c^cre^IL-4Rα^−/lox^ mice. The function of IDO appears to be regulation of tissue inflammation (Rani et al., 2012; Bilir and Sarisozen, 2017). DCs in the CD11c^cre^IL-4Rα^−/lox^ mice expressed RELM-α, another molecule that modulates inflammation (Osborne et al., 2013). IL-4Rα independent expression of RELM-α has been recently reported during hookworm infection (Sutherland et al., 2018), and was upregulated by Ym1, another molecule typically associated with alternative macrophage activation. Previously we have shown that in the absence of IL-4Rα signaling, IL-10 can induce markers of alternative macrophage activation, including Ym1 and mannose receptor (Dewals et al., 2010). Other cytokines, such as TGF-beta and activin A have also been associated with alternative macrophage activation (Ogawa et al., 2006; Gong et al., 2012; Zhang et al., 2016). Further investigations are needed in order to clarify how cytokine signaling regulates classical and alternative activation of DCs and their interaction with intracellular pathogens.

Various lines of evidence support a role for CD11c^+^ cells in replication of *Leishmania* parasites (Leon et al., 2007; De Trez et al., 2009; Hurdayal et al., 2013; Heyde et al., 2018). A recent study by Heyde et al. (2018) described CD11c^+^ cells as monocyte-derived dendritic cell-like, Ly6C^+^CCR2^+^ monocytes (Heyde et al., 2018). These cells were highly infected and harbored parasites with the highest proliferation rate at the site of infection. Depletion of these CD11c-expressing cells resulted in a significant reduction in pathogen burden suggesting that CD11c^+^ cells represent a selective niche for *L. major* proliferation. Our study used a different substrain of *L. major*, but most likely, the cells observed by Heyde et al. were the same subset of inflammatory, monocyte-derived Ly6C^+^ DCs that we observed in our study. In another study, CD11b^+^ CD11c^+^ Ly6C^+^ MHCII^+^ cells were the main infected population in the footpad lesions and draining LNs of *L. major*-infected C57BL/6 mice, and the main iNOS-producing cells (De Trez et al., 2009). Such iNOS-producing inflammatory DC have also been implicated in resistance to *Listeria monocytogenes* and *Brucella melitensis* infection, and have been termed TNF-iNOS-producing DC (TipDCs) or inflammatory DCs (Serbina et al., 2003; Copin et al., 2007; Geissmann et al., 2008). In a visceral leishmaniasis study using *L. donovani*, Ly6C^hi^ inflammatory monocytes formed an important niche for parasite survival, and blocking their recruitment to the liver and spleen using CCR2 antagonists reduced bacterial burdens (Terrazas et al., 2017). In this study, the cells were identified as CD11b^+^Ly6C^hi^Ly6G^−^ and they expressed CD115, CCR2, and intermediate levels of MHCII and F4/80, but they did not express CD11c. Why such cells express CD11c during *L. major* infection but not *L. donovani* infection is not known and remains to be elucidated.

Various markers have been used to identify inflammatory DCs, including Ly6C, F4/80, CD64 (FcγRI), FcεRI, CCR2, DC-SIGN (CD209), and mannose receptor (CD206) (De Trez et al., 2009; Hammad et al., 2010; Langlet et al., 2012; Plantinga et al., 2013; Segura and Amigorena, 2013; Min et al., 2018). CD64 alone is not able to discriminate cDCs and moDCs within CD11b^+^ DCs (Min et al., 2018). In the lung, Ly6C was a specific but insensitive marker for discriminating cDCs and moDCs, since Ly6C expression formed a continuum within CD11b^+^ lung DCs, with Ly6C^low^ cells found to be cDCs and Ly6C^hi^ cells found to be moDCs (Plantinga et al., 2013). The combination of CD64 and FcεRI was suggested as more specific for discriminating moDCs in lung tissue (Plantinga et al., 2013). In our study, we found clear populations of Ly6C^hi^ and Ly6C^lo^ CD11b^+^ DCs containing parasites in the LN and FP, but in the spleen we also found a population of Ly6C^int^ cells. Ly6C^hi^ and Ly6C^int^ CD11b^+^ DC populations were infected with parasites in the spleen, whereas the Ly6C^lo^ CD11b^+^ DC population in the spleen was not. This suggests that the Ly6C^int^ population we observed may also have been moDCs, and suggests that the continuum of Ly6C expression on DCs could be to some extent tissue- and/or disease-specific.

In summary, our data suggest that CD103^+^ DCs play an important role in trafficking parasites to the draining LN at the onset of infection, while later during infection CD11b^+^ DCs become infected. Importantly, dissemination to secondary lymphoid organs, such as the spleen appears to be driven by the migration of CD11b^+^Ly6c^+^ DC inflammatory DCs. In addition, control of parasites in DCs relies on IL-4Rα signaling for early priming of Th1 responses, IFN-γ release and the subsequent upregulation of iNOS. In the absence of IL-4Rα, CD11b^+^Ly6C^+^ DCs provide a safe-haven for parasite replication and dissemination in the infected host. Thus, targeting inflammatory moDC responses may represent a strategy for reducing *L. major* parasite burdens and dissemination.

## Data Availability Statement

The datasets generated for this study are available on request to the corresponding author.

## Ethics Statement

The animal study was reviewed and approved by Faculty of Health Sciences, Research Animal Ethics Committee, University of Cape Town.

## Author Contributions

RH, NN, and FB designed the study. FB provided the gene-deficient mice. RH and NN performed the experiments and analyzed results. RK performed the confocal quantification. RH and FB acquired the funding. RH and NN wrote the original draft. RH, NN, and FB edited the final version of the manuscript. All authors read and approved the final manuscript.

### Conflict of Interest

The authors declare that the research was conducted in the absence of any commercial or financial relationships that could be construed as a potential conflict of interest.

## Supplementary Material

Supplementary Figure 1 CD11c^cre^IL-4Rα^−/lox^ BALB/c are hypersusceptible to cutaneous *Leishmania major* IL81 infection. Mice were infected subcutaneously with 2 × 10^6^ stationary phase virulent GFP-expressing *L. major* IL81 (MHOM/IL/81/FEBNI) parasite strain into the hind footpad. Footpad swelling was measured at weekly intervals. Statistical analysis was performed defining differences to IL-4Rα^−/lox^ mice (^**^, *p* ≤ 0.01) as significant.

Supplementary Figure 2 Representative gating strategy for dendritic cell subsets during *Leishmania major* infection. CD11c^cre^IL-4Rα^−/lox^ BALB/c and control mice were infected subcutaneously with 2 × 10^6^ GFP-labeled *L. major* IL81 promastigotes into the hind footpad. At day 3 or week 4 post-infection, total cells (either footpad, lymph node or spleen) were FACS-stained and gated on FSC-H/FSC-A to obtain singlets. Macrophages and granulocytes were excluded by staining for F4/80 and Ly6G, respectively. The resulting F4/80-Ly6G-negative population was gated for various DC subsets and receptors based on cell-surface markers as depicted.

Supplementary Figure 3 Number of CD11b^+^ dendritic cells is unaltered in CD11c^cre^IL-4Rα^−/lox^ BALB/c and control mice. CD11c^cre^IL-4Rα^−/lox^ BALB/c and control mice were infected subcutaneously with 2 × 10^6^ GFP-labeled *L. major* IL81 promastigotes into the hind footpad. At Day 0, 1, 3, and Week 4 after infection, total spleen cells were stained for CD11c^+^CD11b^+^ dendritic cells by flow cytometry, and total cell numbers enumerated based on spleen cell counts.
