# Supplementary figures and images for: Inflammatory Dendritic Cells, Regulated by IL-4 Receptor Alpha Signaling, Control Replication, and Dissemination of Leishmania major in Mice
Source: Front Cell Infect Microbiol. 2020 Jan 24;9:479. doi: 10.3389/fcimb.2019.00479 (PMC6992597; doi:10.3389/fcimb.2019.00479)

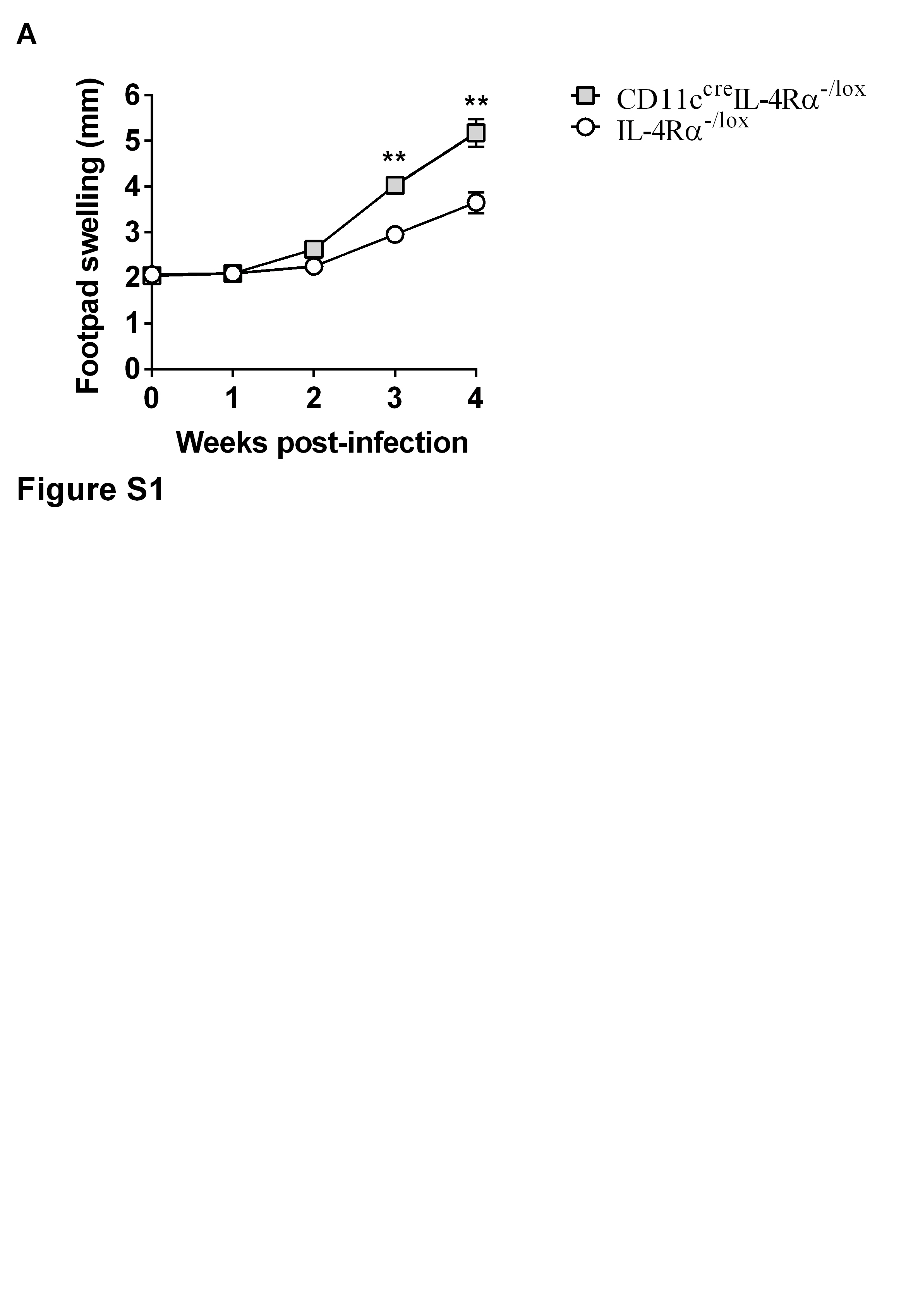

Supplement: Supplementary Figure 1 — CD11ccreIL-4Rα−/lox BALB/c are hypersusceptible to cutaneous Leishmania major IL81 infection. Mice were infected subcutaneously with 2 × 10^6^ stationary phase virulent GFP-expressing L. major IL81 (MHOM/IL/81/FEBNI) parasite strain into the hind footpad. Footpad swelling was measured at weekly intervals. Statistical analysis was performed defining differences to IL-4Rα−/lox mice (**, p ≤ 0.01) as significant. [file Image_1.JPEG]

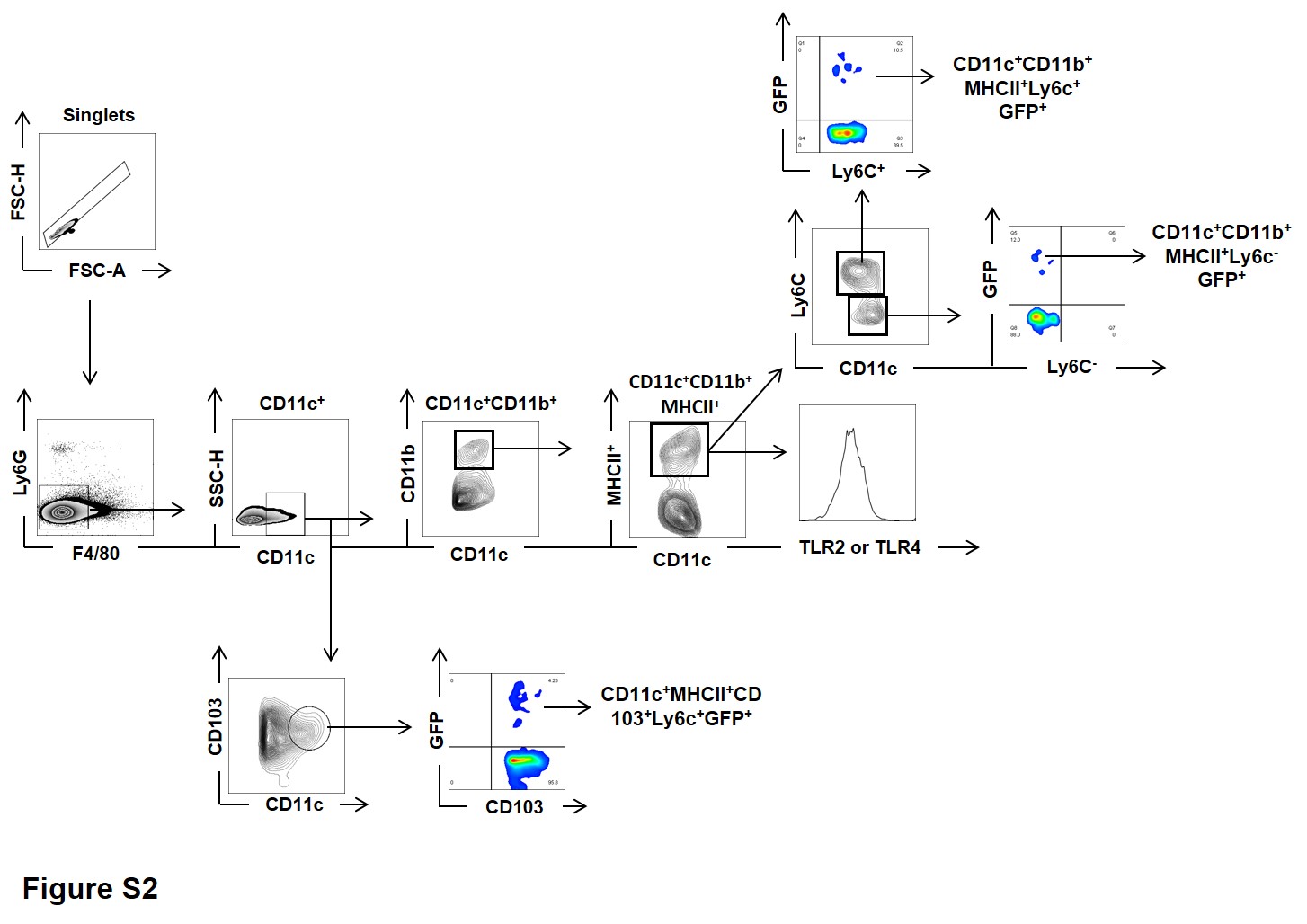

Supplement: Supplementary Figure 2 — Representative gating strategy for dendritic cell subsets during Leishmania major infection. CD11ccreIL-4Rα−/lox BALB/c and control mice were infected subcutaneously with 2 × 10^6^ GFP-labeled L. major IL81 promastigotes into the hind footpad. At day 3 or week 4 post-infection, total cells (either footpad, lymph node or spleen) were FACS-stained and gated on FSC-H/FSC-A to obtain singlets. Macrophages and granulocytes were excluded by staining for F4/80 and Ly6G, respectively. The resulting F4/80-Ly6G-negative population was gated for various DC subsets and receptors based on cell-surface markers as depicted. [file Image_2.JPEG]

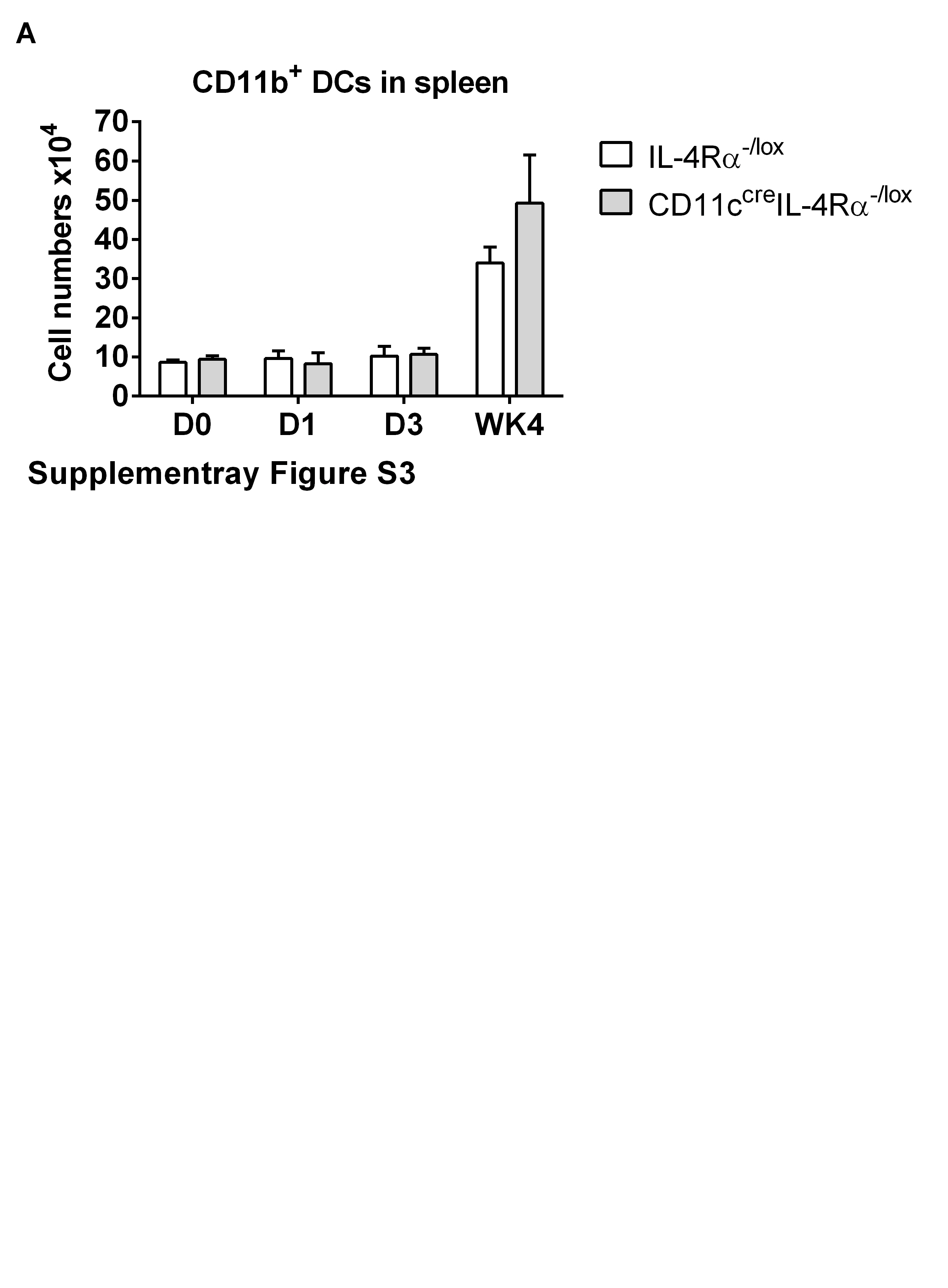

Supplement: Supplementary Figure 3 — Number of CD11b+ dendritic cells is unaltered in CD11ccreIL-4Rα−/lox BALB/c and control mice. CD11ccreIL-4Rα−/lox BALB/c and control mice were infected subcutaneously with 2 × 10^6^ GFP-labeled L. major IL81 promastigotes into the hind footpad. At Day 0, 1, 3, and Week 4 after infection, total spleen cells were stained for CD11c+CD11b+ dendritic cells by flow cytometry, and total cell numbers enumerated based on spleen cell counts. [file Image_3.JPEG]
